# Alveolar Macrophages Are Key Players in the Modulation of the Respiratory Antiviral Immunity Induced by Orally Administered *Lacticaseibacillus rhamnosus* CRL1505

**DOI:** 10.3389/fimmu.2020.568636

**Published:** 2020-09-29

**Authors:** Valeria Garcia-Castillo, Mikado Tomokiyo, Fernanda Raya Tonetti, Md. Aminul Islam, Hideki Takahashi, Haruki Kitazawa, Julio Villena

**Affiliations:** ^1^Laboratory of Immunobiotechnology, Reference Centre for Lactobacilli (CERELA-CONICET), Tucuman, Argentina; ^2^Food and Feed Immunology Group, Laboratory of Animal Products Chemistry, Graduate School of Agricultural Science, Tohoku University, Sendai, Japan; ^3^Livestock Immunology Unit, International Education and Research Center for Food and Agricultural Immunology (CFAI), Graduate School of Agricultural Science, Tohoku University, Sendai, Japan; ^4^Laboratory of Plant Pathology, Graduate School of Agricultural Science, Tohoku University, Sendai, Japan; ^5^Plant Immunology Unit, International Education and Research Centre for Food and Agricultural Immunology (CFAI), Graduate School of Agricultural Science, Tohoku University, Sendai, Japan

**Keywords:** *Lacticaseibacillus rhamnosus* CRL1505, immunobiotics, TLR3, viral immunity, Respiratory Syncytial Virus, alveolar macrophages

## Abstract

The oral administration of *Lacticaseibacillus rhamnosus* CRL1505 differentially modulates the respiratory innate antiviral immune response triggered by Toll-like receptor 3 (TLR3) activation in infant mice, improving the resistance to Respiratory Syncytial Virus (RSV) infection. In this work, by using macrophages depletion experiments and a detailed study of their production of cytokines and antiviral factors we clearly demonstrated the key role of this immune cell population in the improvement of both viral elimination and the protection against lung tissue damage induced by the CRL1505 strain. Orally administered *L. rhamnosus* CRL1505 activated alveolar macrophages and enhanced their ability to produce type I interferons (IFNs) and IFN-γ in response to RSV infection. Moreover, an increased expression of *IFNAR1, Mx2, OAS1, OAS2, RNAseL*, and *IFITM3* was observed in alveolar macrophages after the oral treatment with *L. rhamnosus* CRL1505, which was consistent with the enhanced RSV clearance. The depletion of alveolar macrophages by the time of *L. rhamnosus* CRL1505 administration abolished the ability of infant mice to produce increased levels of IL-10 in response to RSV infection. However, no improvement in IL-10 production was observed when primary cultures of alveolar macrophages obtained from CRL1505-treated mice were analyzed. Of note, alveolar macrophages from the CRL1505 group had an increased production of IL-6 and IL-27 suggesting that these cells may play an important role in limiting inflammation and protecting lung function during RSV infection, by increasing the maturation and activation of Treg cells and their subsequent production of IL-10. In addition, we provided evidence of the important role of CD4^+^ cells and IFN-γ in the activation of alveolar macrophages highlighting a putative pathway through which the intestinal and respiratory mucosa are communicated under the influence of *L. rhamnosus* CRL1505.

## Introduction

The effect of the intestinal microbiota on the immune responses in the respiratory tract and its impact on the outcome of viral infections has been explored during the last decade (1–3). Those studies demonstrated that the signals provided by the intestinal microbiota act at multiple levels in the respiratory mucosa stimulating an antiviral state in non-immune cells and innate immune cells that would allow an efficient control of viral replication early during the infection. Although these effects of the intestinal microbiota have been documented mainly for Influenza Virus (IFV) infection (1–3), it should be considered that the cellular and molecular mechanisms involved in the innate antiviral immune response are not virus specific and therefore, they are similar for all the respiratory viruses. Then, the beneficial microbes in the intestinal tract may favorably influence the innate immune responses to other respiratory viruses as well. In this regard, it was recently demonstrated that a high-fiber diet improved the production of acetate by the intestinal microbiota of mice and that this metabolic product modulated the activity of respiratory interferon (IFN)-β and increased the expression of interferon-stimulated genes (ISGs) in the lung (4). The immunological changes induced in the respiratory tract by the dietary treatment significantly increased the resistance of mice to the challenge with Respiratory Syncytial Virus (RSV).

The studies with germ-free as well as antibiotic-treated mice recolonized with bacteria demonstrated that the changes induced by the intestinal microbiota in the respiratory antiviral immune response are reversible and more importantly, tunable (1–3, 5). Moreover, it was shown that not all commensal bacteria could contribute equally to the antiviral immunocompetence in the lung (1). These findings have opened the possibility of exploring particular strains of beneficial bacteria with immunomodulatory capacities, referred to as immunobiotics, in order to increase antiviral defenses in the respiratory tract. Then, several immunomodulatory beneficial microbial strains mainly from the Lactobacillus and Bifidobacterium species; have been tested in their capacities to modulate the respiratory antiviral immune response when orally administered (6, 7). A growing number of studies have examined the effect of immunobiotic nutritional interventions on the incidence, the duration and severity of respiratory infections in humans. Several clinical trials, systematic reviews and meta-analyses have suggested that immunobiotics may be effective in improving the resistance of children against viral respiratory infections such as the common cold and influenza-like symptoms (8, 9). Interestingly, immunobiotic intervention was shown to reduce the episodes of viral respiratory infections even in asthmatic children (10). Collectively, research has shown that some immunobiotic strains are capable of making a difference in the host's response to respiratory viral infections. However, there is limited information regarding the cellular and molecular mechanisms involved in the beneficial effects of each particular immunobiotic strain. Those kind of studies are necessary to provide solid scientific basis to promote the use of immunobiotics in the prevention of respiratory viral infections.

In a randomized controlled trial in children, it was demonstrated that the immunobiotic strain *Lacticaseibacillus rhamnosus* CRL1505 (Basonym, *Lactobacillus rhamnosus* CRL1505) (11), administered in a yogurt, improved mucosal immunity and reduced the incidence and severity of viral intestinal and respiratory infections (12). Since this finding in children, our laboratory had perform studies by using *in vivo* and *in vitro* models to characterize the antiviral properties of *L. rhamnosus* CRL1505. We demonstrated that the oral administration of the CRL1505 strain was capable of improving the resistance of infant mice to RSV infection (13, 14). We found that *L. rhamnosus* CRL1505 differentially regulated the respiratory innate antiviral immune response triggered by the activation of Toll-like receptor 3 (TLR3), improving the resistance to RSV infection. The study of the immunological mechanisms involved in the protective effect induced by *L. rhamnosus* CRL1505 revealed a key role for respiratory IFN-β, IFN-γ, and interleukin (IL)-10. Our results showed that the increase of the three cytokines in the respiratory tract induced by the oral CRL1505 treatment was involved in the reduction of lung RSV titers and inflammatory-mediated lung tissue injury. Moreover, our studies provided us with preliminary information that indicate that CD11c^+^SiglecF^+^ alveolar macrophages would actively participate in the beneficial effects induced by *L. rhamnosus* CRL1505 (13, 14). However, the exact role of resident alveolar macrophages in the immunomodulatory effects of the CRL1505 strain have not been investigated in detail.

Considering this background, in this work we aimed to further advance in the characterization of the beneficial effects of *L. rhamnosus* CRL1505 in the context of respiratory RSV infection by evaluating whether their immunomodulatory properties are dependent on alveolar macrophages function. The role of alveolar macrophages in the differential cytokine profile induced in the respiratory tract by orally administered *L. rhamnosus* CRL1505, as well as in its ability to increase the resistance to RSV infection was evaluated.

## Materials and Methods

### Microorganisms

*Lacticaseibacillus rhamnosus* CRL1505 and *Lactiplantibacillus plantarum* CRL1506 (Basonym, *Lactobacillus plantarum* CRL1505) (11) were obtained from the CERELA culture collection (Chacabuco 145, San Miguel de Tucumán, Argentina). Both lactobacilli cultures were kept freeze-dried. Lactobacilli were cultured for 12 h at 37°C (final log phase) in Man-Rogosa-Sharpe broth (MRS, Oxoid). The bacteria were harvested by centrifugation at 3,000 g for 10 min, washed three times with sterile 0.01 mol/L phosphate buffer saline (PBS, pH 7.2), and resuspended in sterile 10% non-fat milk.

### Animals and Treatments

Infant (3-week-old) BALB/c mice were obtained from the closed colony kept at CERELA (San Miguel de Tucumán, Argentina). Animals were housed in plastic cages at room temperature and the assays for each parameter studied were performed in 5–6 mice per group for each time point. Three groups of mice were used: CRL1505- and CRL1506-treated mice and PBS-treated controls. *L. rhamnosus* CRL1505 and *L. plantarum* CRL1506 strains were orally administered to infant mice for five consecutive days. Mice were deprived of water for 4 h and the immunobiotic strains were given at a dose of 10^8^ cells/mouse/day in a minimum volume of drinking water containing 10% of non-fat milk to animals in individual cages (13, 14). The treated groups and the PBS-treated control group were fed a conventional balanced diet *ad libitum*.

This study was carried out in strict accordance with the recommendations in the Guide for the Care and Use of Laboratory Animals of the Guidelines for Animal Experimentation of CERELA. The CERELA Institutional Animal Care and Use Committee prospectively approved this research under the protocol BIOT-CRL-18. All efforts were made to minimize the number of animals and their suffering. No signs of discomfort or pain were observed before mice reached the endpoints. No deaths were observed before mice reached the endpoints.

Blood and respiratory tissue samples were obtained from mice after the intraperitoneal injection of ketamine (80 mg/kg) and xylazine (10 mg/kg) according to the recommendations of the CERELA Institutional Animal Care and Use Committee.

### Poly(I:C) Administration and RSV Infection

Administration of the TLR3 agonist poly(I:C) (Sigma-Aldrich) was performed 2 days after the last day of lactobacilli treatments. Mice received 100 μl of PBS containing 250 μg poly(I:C) (equivalent to 10 mg/kg body weight), that was administered dropwise, via the nares (13, 15, 16). Control animals received 100 μl of PBS. Mice received three doses of poly (I:C) or PBS with 24 h rest period between each administration.

Human RSV strain A2 was grown in Vero cells as described previously (13–15). Briefly, Vero cells were grown in Dulbecco's modified Eagle's medium (DMEM) and infected with RSV at a multiplicity of infection (MOI) of 1 in 5 ml for 3 h at 37°C, 5% CO_2_. After infection, 7 ml of DMEM with 10% fetal bovine serum (Sigma, Tokyo, Japan), 0.1% penicillin-streptomycin (Pen/Strep) (Sigma, Tokyo, Japan), and 0.001% ciprofloxacin (Bayer) was added to the flask, and cells were incubated until extensive syncytium formation was detected. Then, Vero cells were scraped, sonicated and cell debris was removed by centrifugation at 700 g for 10 min at 4°C. Virus supernatant was sucrose density gradient purified and stored in 30% sucrose at −80°C. For *in vivo* infection, mice were challenged with 10^6^ PFU (Plaque forming units) of RSV by the nasal route (13–15). Viral challenge was performed 2 days after the last day of lactobacilli treatments. Lung RSV titers and tissue damage were evaluated 2 days after viral infection. The RSV immunoplaque assay was performed as described previously (14–16), briefly lungs were homogenized and diluted tissue clarified supernatants were added to Vero cells monolayers. Samples were run in triplicate. After 3 h of incubation (37°C, 5% CO_2_) supernatants were removed and fresh medium (DMEM, 10% FBS, 0.1% Pen- Strep, 0.001% ciprofloxacin) was added. Monolayers were fixed with ice cold acetone: methanol (60:40) when extensive syncytia were observed. Primary RSV anti-F (clones 131-2A; Chemicon), anti-G (Mouse monoclonal [8C5 (9B6)] to RSV glycoprotein, Abcam) and secondary horseradish peroxidase anti-mouse immunoglobulin (Anti-mouse IgG, HRP-linked Antibody #7076, Cell signaling Technology) antibodies were used. Individual plaques were developed using a DAB substrate kit (ab64238, Abcam) following manufacture's specifications. Results were expressed as log_10_ PFU/g of lung.

### Lung Injury Parameters

Broncho-alveolar lavages (BAL) samples were obtained as described previously (16, 17). Briefly, the trachea was exposed and intubated with a catheter, and 2 sequential lavages were performed in each mouse by injecting sterile PBS. The recovered fluid was centrifuged for 10 min at 900 g; and frozen at −70°C for subsequent analyses.

Albumin content, a measure to quantitate increased permeability of the bronchoalveolar–capillarity barrier, and lactate dehydrogenase (LDH) activity, an indicator of general cytotoxicity, were determined in the acellular BAL fluid. Albumin content was determined colorimetrically based on albumin binding to bromcresol green using an albumin diagnostic kit (Wiener Lab, Buenos Aires, Argentina). LDH activity, expressed as units per liter of BAL fluid, was determined by measuring the formation of the reduced form of nicotinamide adenine dinucleotide (NAD) using the Wiener reagents and procedures (Wiener Lab).

### *In vivo* Depletion of Alveolar Macrophages

**Figure 1:**
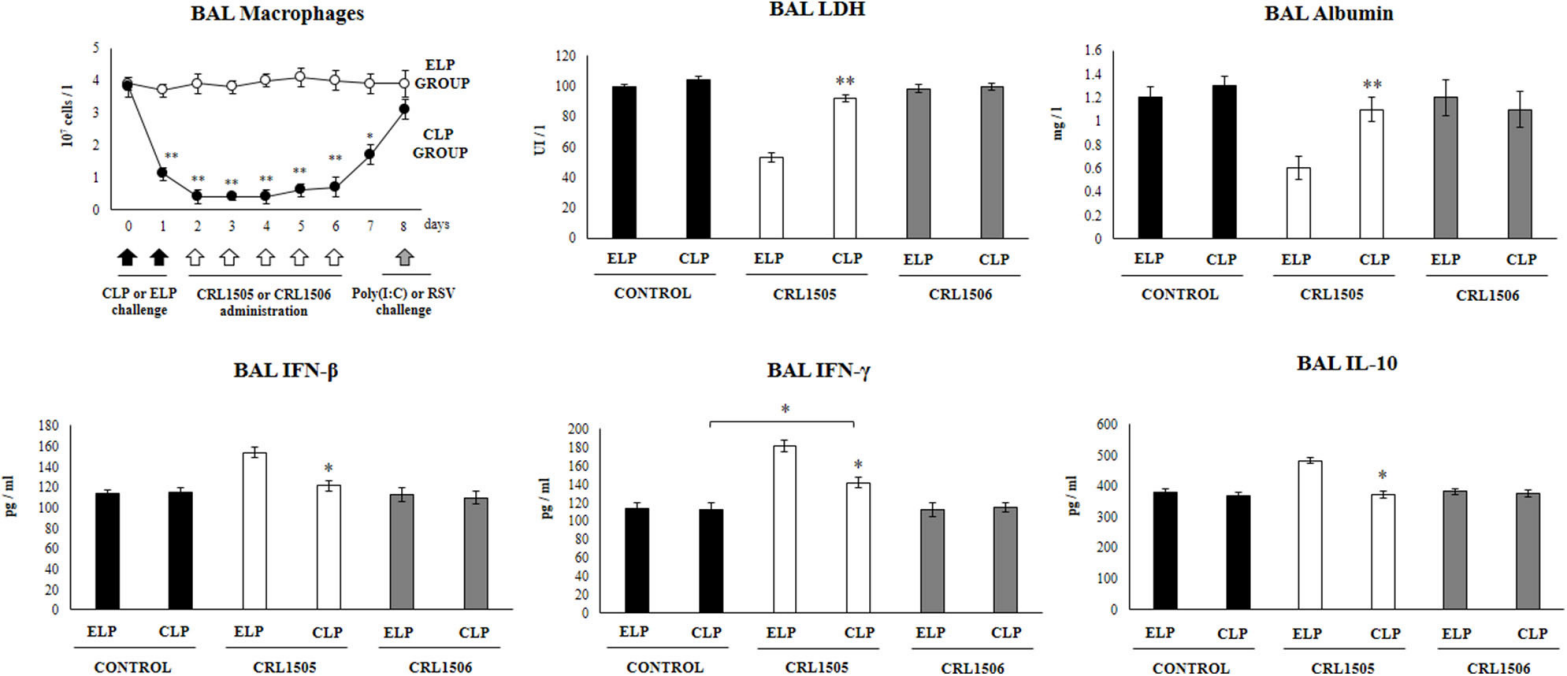
Effect of alveolar macrophages depletion on the ability of *Lacticaseibacillus rhamnosus* CRL1505 to modulate the respiratory immune response triggered by poly(I:C) treatment. Infant mice were nasally treated with clodronate-containing liposomes (CLP) during 2 days (days 0 and 1) to induce the depletion of alveolar macrophages. One day after the last CPL administration mice were orally treated with *L. rhamnosus* CRL1505 or *Lactiplantibacillus plantarum* CRL1506 (10^8^ cell/mouse/day) during five consecutive days (days 2–6) and challenged with three once-daily doses of poly(I:C) (days 8, 9, and 10). Mice treated with empty liposomes (ELP), CRL1505 or CRL1506 and then challenged with poly(I:C) were used as controls. Two days after the last poly(I:C) administration lactate dehydrogenase (LDH) activity, albumin concentrations, and the levels of interferon (IFN)-β, IFN-γ, and interleukin (IL)-10 in broncho-alveolar lavages (BAL) were evaluated. The results represent data from three independent experiments. Asterisks indicate significant differences between the respective ELP and CLP groups. Asterisks in black lines indicate significant differences between the indicated groups. *P < 0.05, **P < 0.01.

For depletion of alveolar macrophages, mice were inoculated intranasally with 50 μl of clodronate (dichloromethylene-bisphosphonate)-containing liposomes (CLP; Clophosome, Stratech, United Kingdom) as described elsewhere (18). Mice were treated with CLP for two consecutive days. Optimal conditions of alveolar macrophages depletion were determined by differential counting of BAL cells (Figure 1). An equal treatment with empty liposomes (ELP) served as controls.

The total number of leukocytes and differential cell counts in BAL samples were performed as described previously (13, 15, 16). Briefly, the total number of leukocytes was determined with a hemocytometer. Differential cell counts were performed by counting 200 cells in blood smears stained with May Grunwald-Giemsa.

### Alveolar Macrophages Primary Cultures

Primary cultures of murine alveolar macrophages was performed as described elsewhere (19, 20). Macrophages were obtained from infant mice via bronchoalveolar lavages by using 1 ml of warm sterile PBS containing 5 mM EDTA. Macrophages were transferred to new sterile tubes, washed twice in sterile PBS, and resuspended in RPMI 1640 medium with 10% FBS, 1 mM L-glutamine, and 100 U/ml penicillin-streptomycin. BAL cells were seeded in 24-well-plates at a density of 10^5^ cells/well and incubated for 2 h at 37°C in 5% CO_2_ to promote adherence. Non-adherent cells were washed and macrophages were maintained in culture in RPMI 1640 medium with 10% FBS, 1 mM L-glutamine, and 100 U/ml penicillin-streptomycin at 37°C in 5% CO_2_ for 24 h before stimulation. Alveolar macrophages were stimulated with poly(I:C) (50 ug/ml), or RSV (MOI of 5). Supernatants were collected before (basal conditions) and 24 h after stimulations, for cytokines analysis. In addition, the mRNA was extracted from alveolar macrophages 12 h after RSV challenge for the evaluation of cytokines and antiviral factors gene expressions.

### Cytokine Concentrations in BAL and Culture Supernatants

BAL samples were obtained as described previously (16, 17). The samples were frozen at −70°C for subsequent cytokine analyses. IFN-β (Mouse IFN-beta ELISA Kit, sensitivity: 15.5 pg/ml), IFN-γ (Mouse IFN-gamma Quantikine ELISA Kit, sensitivity: 2 pg/ml), IL-6 (Mouse IL-6 Quantikine ELISA Kit, sensitivity: 1.8 pg/ml), IL-10 (Mouse IL-10 Quantikine ELISA Kit, sensitivity: 5.2 pg/ml), IL-12 (Mouse IL-12 p70 DuoSet ELISA, sensitivity: 1.5 pg/ml) and IL-27 (Mouse IL-27 p28/IL-30 Quantikine ELISA Kit, sensitivity: 4.7 pg/ml), IL-17 (Mouse IL-17 Quantikine ELISA Kit, sensitivity 5 pg/ml), and the IL-8 mouse homolog chemokine KC or chemokine (C-X-C motif) ligand 1 (Mouse CXCL1/KC DuoSet ELISA, sensitivity 2.3 pg/ml) concentrations in BAL and culture supernatants samples were measured with commercially available enzyme-linked immunosorbent assay (ELISA) technique kits following the manufacturer's recommendations (R&D Systems, MN, USA).

### Quantitative Expression Analysis by Real-Time PCR

Alveolar macrophages were obtained as described above and total RNA was isolated from each sample using TRIzol reagent (Invitrogen) according to the manufacturer's instructions. After the chloroform step, the aqueous phase–containing RNA was further processed using the RNeasy Micro kit (QIAGEN) according to the manufacturer's instructions.

Two-step real-time quantitative PCR was performed to characterize the expression of *IFN-*α*, IFN-*β*, IFN-*γ, IFN-alpha/beta receptor alpha chain (*IFNAR1*)*, IFN-*λ*1, IFN-*λ*2/3*, the IFN-induced GTP-binding protein Mx1 (*Mx1*)*, Mx2*, the 2′-5′-oligoadenylate synthetase 1 (*OAS1*)*, OAS2*, the ribonuclease L (*RNAseL*), and the IFN-induced transmembrane protein 3 (*IFITM3*) genes in cultured alveolar macrophages. All cDNAs were synthesized using a Quantitect reverse transcription (RT) kit (Qiagen, Tokyo, Japan) according to the manufacturer's recommendations. Real-time quantitative PCR was carried out using a 7300 real-time PCR system (Applied Biosystems, Warrington, United Kingdom) and the Platinum SYBR green qPCR SuperMix uracil-DNA glycosylase (UDG) with 6-carboxyl-X-rhodamine (ROX) (Invitrogen). The primers used in this work are given in the (Supplementary Table 1). The PCR cycling conditions were 2 min at 50°C, followed by 2 min at 95°C, and then 40 cycles of 15 s at 95°C, 30 s at 60°C, and 30 s at 72°C. The reaction mixtures contained 5 μl of sample cDNA and 15 μl of master mix, which included the sense and antisense primers. Expression of β-actin was used to normalize cDNA levels for differences in total cDNA levels in the samples.

### Flow Cytometry Analysis

Single cells from BAL samples were prepared as previously described (14, 16, 17). Erythrocytes were depleted by hypotonic lysis and the cells were washed with RPMI medium supplemented with 10% heat-inactivated fetal calf serum (FCS). Cells were counted using Trypan Blue exclusion and then resuspended at an appropriate concentration of 5 × 10^6^ cells/ml.

BAL cell suspensions were pre-incubated with anti-mouse CD32/CD16 monoclonal antibody (Fc block) for 15 min at 4°C. Cells were incubated in the antibody mixes for 30 min at 4°C and washed with FACS buffer. Then, cells were stained with fluorochrome-conjugated antibodies against CD11c (APC), SiglecF (PE) (BD Bioscience), CD45 (FITC) (eBioscience), and MHC-II (PerCP) (Thermo Fisher Scientific). Cells were then acquired on a BD FACSCalibur™ flow cytometer (BD Biosciences) and data were analyzed with FlowJo software (TreeStar). The total number of cells in each population was determined by multiplying the percentages of subsets within a series of marker negative or positive gates by the total cell number determined for each BAL sample (14, 16, 17).

### Blocking Experiments

In order to evaluate the role of IFN-γ in the immunomodulatory capacity *L. rhamnosus* CRL1505, anti-IFN-γ blocking antibodies were used. Different groups of mice were orally treated with *L. rhamnosus* CRL1505 for 5 consecutive days at a dose of 10^8^ cells/mouse/day as described above. On days 1, 3, and 5 mice were injected intraperitoneally with 20 ug of purified anti-IFN-γ antibodies (LEAF™ Purified anti-mouse IFN-γ IgG1 antibody, #505706 BioLegend) or 80 ug of isotype control antibodies (LEAF™ Purified Rat IgG1, % Isotype Ctrl, LEAF™ Purified Rat IgG1, % Isotype Ctrl, BioLegend) as we described previously (13). For CD4 depletion, mice were intraperitoneally injected with 200 μg of anti-α-CD4 antibodies (Rat IgG2b, clone GK1.5; #AB1107636, BioXcell) or IgG isotype control antibodies (rat IgG2b isotype, clone LTF-2, #BE0090, BioXcell) on days 1, 3, and 5.

### Statistical Analysis

Experiments were performed in triplicate and results were expressed as mean ± standard deviation (SD). After verification of the normal distribution of data, 2-way ANOVA was used. Tukey's test (for pairwise comparisons of the means) was used to test for differences between the groups. Differences were considered significant at *p* < 0.05.

## Results

### Effect of Alveolar Macrophages Depletion on the Capacity of *L. rhamnosus* CRL1505 to Modulate the Respiratory Immune Response Triggered by Poly(I:C)

The nasal administration of liposomes containing toxic substances such as clodronate is a widely used technic to evaluate the role of alveolar macrophages in respiratory immune responses (18, 20). Here, we used this experimental approach to evaluate the role of alveolar macrophages in the immunomodulatory effects of orally administered *L. rhamnosus* CRL1505. In addition, we used the immunobiotic strain *L. plantarum* CRL1506 for comparison. Our previous studies demonstrated that the oral administration of the CRL1505 or CRL1506 strains to mice are able to beneficially modulate intestinal antiviral immunity (21, 22). However, only *L. rhamnosus* CRL1505 exerts a beneficial effect at a distance by modulating respiratory immunity.

In our hands, the nasal administration of clodronate-containing liposomes (CLP) significantly reduced the number of macrophages in BAL samples for a period of 6 days. The number of BAL macrophages started to recover from day 7 (Figure 1). This effect was not observed in infant mice nasally treated with empty liposomes (ELP) in which BAL macrophages numbers were normal during all the assessed period. These results indicated that CLP treatment is useful for evaluating the role of alveolar macrophages in the immunomodulatory effect of the CRL1505 strain in our experimental models, since these immune cells are decreased at the time of lactobacilli administration, while their number return to normality when the poly(I:C) or RSV challenges occur.

We and others demonstrated that the nasal administration of the TLR3 agonist poly(IC) to mice induce an inflammatory response and lung functional changes that are similar to those caused by RSV (13, 14). Therefore, we used the nasal administration of the dsRNA analog poly(I:C) to mimic the pro-inflammatory and physiopathological consecuences of RNA viral infections in the lung. In addition, we used the BAL biochemical markers albumin and LDH to assess the lung damage. Albumin is not detected normally in BAL samples and the increase of this protein in the respiratory tract is an indicator of an increased permeability of the bronchoalveolar–capillarity barrier. On the other hand, the increase of the intracellular enzyme LDH in BAL samples is an indicator of cytotoxicity (13, 14).

It was observed that in infant mice treated with ELP and challenged with poly(I:C) the levels of BAL LDH and albumin increased significantly after TLR3 activation (Figure 1). It was also observed that in animals treated with ELP and *L. rhamnosus* CRL1505 and subsequently challenged with poly(I:C) the levels of BAL LDH and albumin were significantly lower than those found in their respective control group (ELP control mice) (Figure 1). These results clearly indicate that treatment with ELP does not modify the number or functionality of alveolar macrophages, since both the effect of poly(I:C) and the CRL1505 strain in ELP-treated mice are similar to those previously described in conventional mice (13, 14).

The administration poly(I:C) to CLP-treated control mice significantly increased the values of lung damage markers with no differences respect to ELP control animals. It was also observed that in infant mice treated with CLP, the administration of *L. rhamnosus* CRL1505 was not able to reduce the levels of BAL LDH and albumin after the challenge with poly(I:C) (Figure 1). Mice treated with ELP or CLP and *L. plantarum* CRL1506 showed lung injury markers values that were not different from their respective controls (Figure 1).

The levels of IFN-β, IFN-γ, and IL-10 in BAL were also determined after administration of poly(I:C), in infant mice treated with CLP or ELP (Figure 1). The administration of the TLR3 agonist induced significant increases in the levels of BAL IFN-β, IFN-γ, and IL-10 in the ELP control mice, which were similar to those previously reported (13, 14). It was also observed that *L. rhamnosus* CRL1505 induced significant increases in the levels of BAL IFN-β and IFN-γ as well as in IL-10 after the administration of poly(I:C) in ELP-treated mice compared with ELP controls (Figure 1).

In control infant mice treated with CLP, the values of the three cytokines were similar to the found in the ELP control group after the stimulation with poly(I:C). The treatment of CLP mice with *L. rhamnosus* CRL1505 increased the levels of IFN-γ, but the values of this cytokine did not reach the levels observed in mice treated with ELP and the CRL505 strain (Figure 1). It was also observed that in infant mice treated with CLP, the administration of *L. rhamnosus* CRL1505 was not able to increase the levels of BAL IFN-β or IL-10 after the challenge with poly(I:C) when compared to the CLP control group (Figure 1). Again, mice treated with ELP or CLP and *L. plantarum* CRL1506 showed respiratory cytokines values that were not different from their respective controls (Figure 1).

These results suggest that alveolar macrophages are a key immune cell population involved in the differential cytokine induction and in the protection against lung damage induced by orally administered *L. rhamnosus* CRL1505 in the context of TLR3-mediated inflammation.

### Effect of Alveolar Macrophages Depletion on the Capacity of *L. rhamnosus* CRL1505 to Modulate the RSV Infection

**Figure 2:**
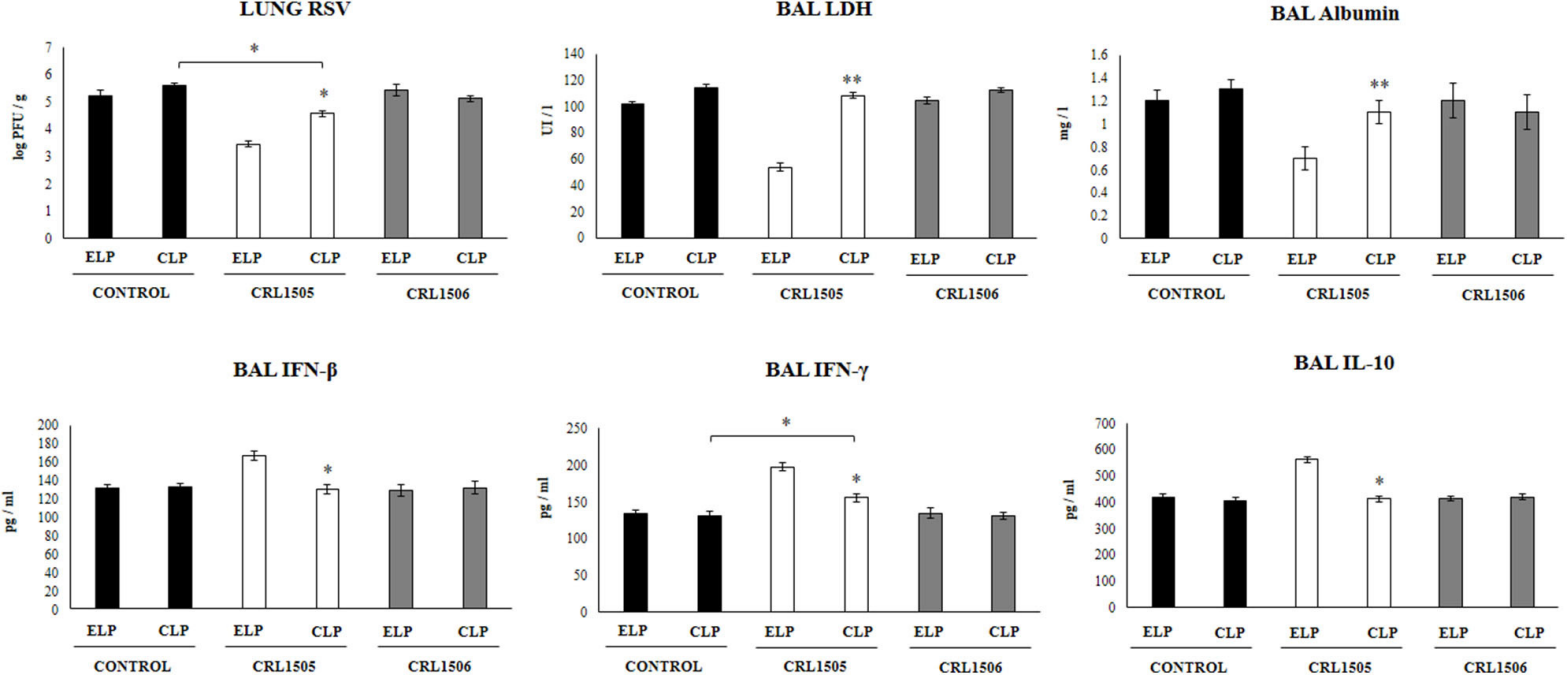
Effect of alveolar macrophages depletion on the ability of *Lacticaseibacillus rhamnosus* CRL1505 to modulate the respiratory immune response triggered by Respiratory Syncytial Virus (RSV) infection. Infant mice were nasally treated with clodronate-containing liposomes (CLP) during 2 days (days 0 and 1) to induce the depletion of alveolar macrophages. One day after the last CPL administration mice were orally treated with *L. rhamnosus* CRL1505 or *Lactiplantibacillus plantarum* CRL1506 (10^8^ cell/mouse/day) during five consecutive days (days 2–6) and then challenged with RSV (day 8). Mice treated with empty liposomes (ELP), CRL1505 or CRL1506 and then challenged with poly(I:C) were used as controls. Two days after the viral challenge, lung RSV titers, lactate dehydrogenase (LDH) activity, albumin concentrations, and the levels of interferon (IFN)-β, IFN-γ, and interleukin (IL)-10 in broncho-alveolar lavages (BAL) were evaluated. The results represent data from three independent experiments. Asterisks indicate significant differences between the respective ELP and CLP groups. Asterisks in black lines indicate significant differences between the indicated groups. *P < 0.05, **P < 0.01.

We further evaluated the impact of alveolar macrophages depletion in the ability of *L. rhamnosus* CRL1505 to improve the resistance against RSV infection. As shown in Figure 2, infant mice treated with CLP had similar lung RSV titers than the observed in ELP-treated mice. The *L. rhamnosus* CRL1505 treatment was able to reduce RSV titers in both ELP- and CLP-treated infant mice. However, the lung viral titers in CLP+CRL1505 mice were significantly higher than the found in the ELP+CRL1505 group (Figure 2). It was also observed that *L. rhamnosus* CRL1505 was able to significantly reduce BAL albumin and LDH values in ELP-treated infant mice after the infection with RSV (Figure 2). In CLP-treated mice, the levels of BAL albumin and LDH were similar to the infant mice in the ELP control group. *L. rhamnosus* CRL1505 was not able to reduce the levels of BAL albumin or LDH in CLP-treated infant mice when compared to the CLP control group (Figure 2). Mice treated with ELP or CLP and *L. plantarum* CRL1506 showed lung injury markers values that were not different from their respective controls after the challenge with RSV (Figure 2).

The levels of BAL IFN-β, IFN-γ, and IL-10 were also determined after the RSV infection in infant mice treated with liposomes (Figure 2). The viral challenge significantly augmented the levels of BAL IFN-β, IFN-γ, and IL-10 in ELP-treated and CLP-treated mice, with no differences between the groups. Similar to our previous results (13), it was observed that *L. rhamnosus* CRL1505 significantly augmented the levels of BAL IFN-β, IFN-γ, and IL-10 after RSV challenge of infant mice treated with ELP. The levels of both BAL IFN-β and IFN-γ in mice treated with CLP+CRL1505 were significantly lower than the found in the ELP+CRL1505 group (Figure 2). However, BAL IFN-γ in the CLP+CRL1505 group was significantly higher than CLP controls. In addition, it was observed that in infant mice that received CLP, the treatment with *L. rhamnosus* CRL1505 was unable to modify BAL IL-10 values after RSV infection when compared to the CLP control group (Figure 2). Mice treated with ELP or CLP and *L. plantarum* CRL1506 showed respiratory cytokines values that were not different from their respective controls (Figure 2).

These RSV challenge experiments suggest that alveolar macrophages are a key immune cell population involved in the protection against virus-induced lung damage as well as in the differential respiratory cytokine profile induced by orally administered *L. rhamnosus* CRL1505.

### Effect of *L. rhamnosus* CRL1505 on Alveolar Macrophages Cytokine Profiles in Response to Poly(I:C) or RSV

**Figure 3:**
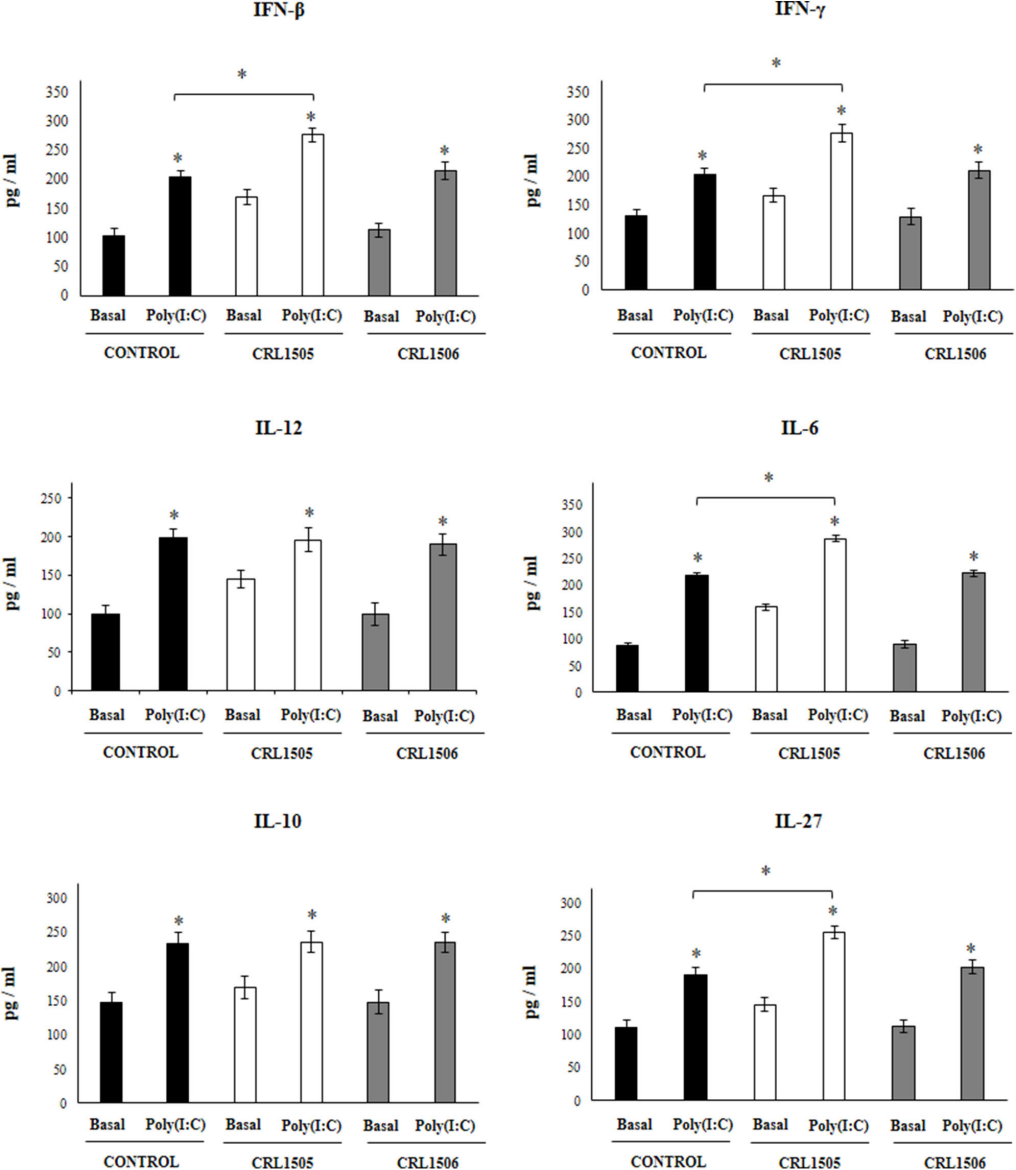
Effect of *Lacticaseibacillus rhamnosus* CRL1505 on alveolar macrophages cytokine profiles in response to poly(I:C) challenge. Infant mice were orally treated with *L. rhamnosus* CRL1505 or *Lactiplantibacillus plantarum* CRL1506 (10^8^ cell/mouse/day) during five consecutive days. One day after the last lactobacilli administration, alveolar macrophages were isolated from infant mice, cultured, and challenged *in vitro* with poly(I:C). Twenty-four hours after the poly(I:C) stimulation, the levels of interferon (IFN)-β, IFN-γ, interleukin (IL)-6, IL-10, IL-12, and IL-27 were evaluated on alveolar macrophages supernatants. The results represent data from three independent experiments. Asterisks indicate significant differences between the basal and post-poly(I:C) challenge time points within each group. Asterisks in black lines indicate significant differences between the indicated groups. *P < 0.05.

**Figure 4:**
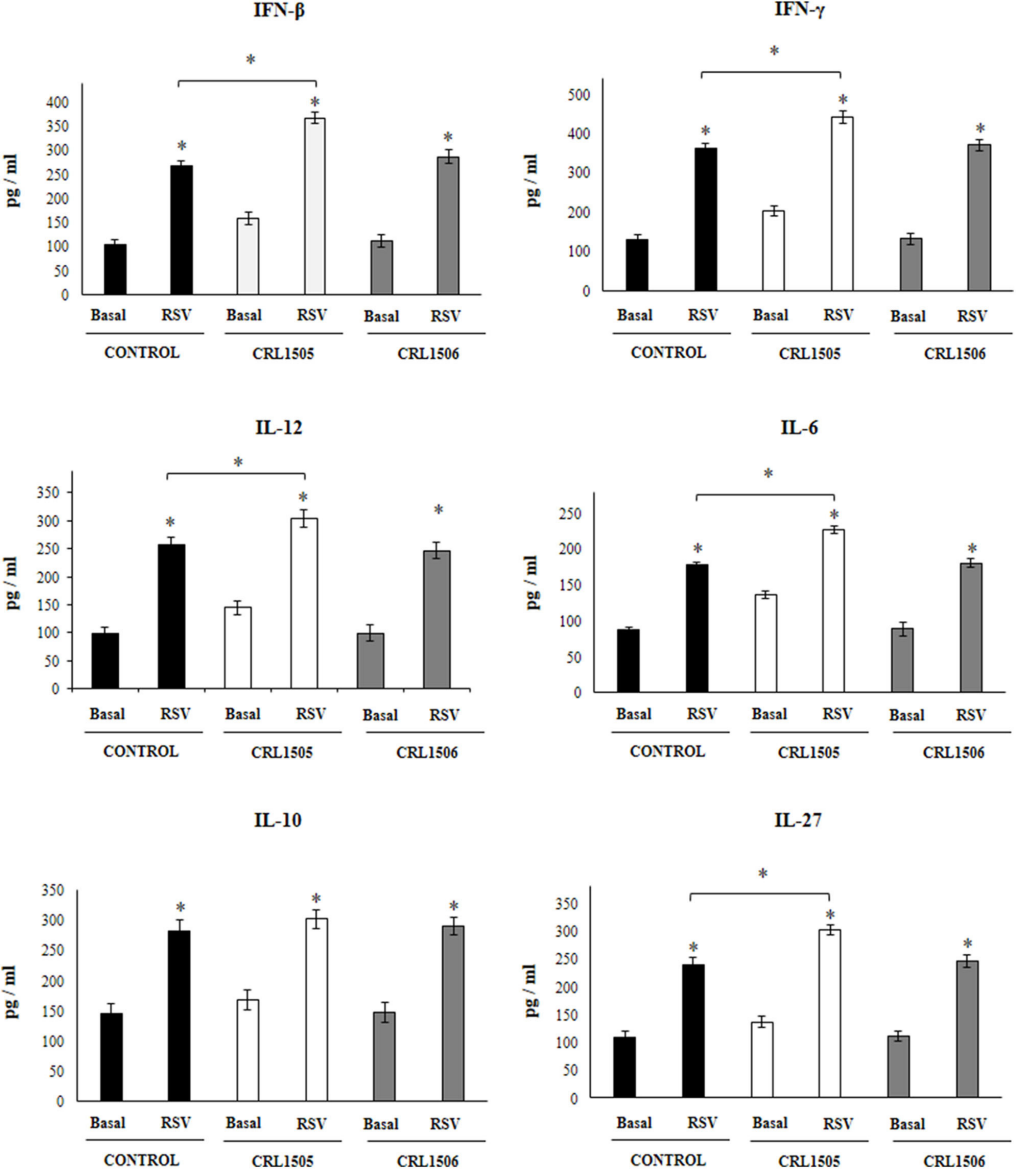
Effect of *Lacticaseibacillus rhamnosus* CRL1505 on alveolar macrophages cytokine profiles in response to Respiratory Syncytial Virus (RSV) infection. Infant mice were orally treated with *L. rhamnosus* CRL1505 or *Lactiplantibacillus plantarum* CRL1506 (10^8^ cell/mouse/day) during five consecutive days. One day after the last lactobacilli administration, alveolar macrophages were isolated from infant mice, cultured and challenged *in vitro* with RSV. Twenty-four hours after the viral infection, the levels of interferon (IFN)-β, IFN-γ, interleukin (IL)-6, IL-10, IL-12, and IL-27 were evaluated on alveolar macrophages supernatants. The results represent data from three independent experiments. Asterisks indicate significant differences between the basal and post-RSV challenge time points within each group. Asterisks in black lines indicate significant differences between the indicated groups. *P < 0.05.

Taking into consideration that the previous results suggested that the alveolar macrophages would have a relevant role in the immunomodulatory effect of *L. rhamnosus* CRL1505, the changes induced by the strain on alveolar macrophages cytokine profile were then studied. For this purpose, primary cultures of alveolar macrophages from control, *L. rhamnosus* CRL1505- or *L. plantarum* CRL1506-treated infant mice were performed and cells were challenged *in vitro* with poly(I:C) (Figure 3), or RSV (Figure 4). Basal production of IFN-β, IFN-γ, IL-6, IL-12 as well as the immunoregulatory cytokines IL-10 and IL-27 was detected in alveolar macrophages cultures. Moreover, the basal levels of all the cytokines evaluated were significantly higher in alveolar macrophages cultures obtained from *L. rhamnosus* CRL1505-treated infant mice when compared to controls (Figure 3). On the contrary, the levels of all the cytokines evaluated in alveolar macrophages cultures obtained from *L. plantarum* CRL1506-treated infant mice were not different from controls (Figure 3).

The challenge with poly(I:C) (Figure 3) significantly increased the levels of IFN-β, IFN-γ, IL-6, and IL-12 in control alveolar macrophages cultures as well as in those obtained from CRL1505- or CRL1506-treated infant mice. However, the concentrations of IL-6, IFN-β, and IFN-γ were significantly higher in alveolar macrophages cultures from *L. rhamnosus* CRL1505-treated infant mice when compared to the control group, while IL-12 was not different from control macrophages. In addition, poly(I:C) challenge significantly increased the levels of IL-10 and IL-27 in alveolar macrophages cultures. However, the concentrations of IL-27 were significantly higher in cultures from *L. rhamnosus* CRL1505-treated infant mice when compared to the control group. No differences were observed in IL-10 levels when control macrophages were compared to those obtained from CRL1505-treated infant mice (Figure 3). Similarly, the challenge with RSV significantly increased IFN-β, IFN-γ, IL-6, IL-12, IL-10, and IL-27 in all the experimental groups. However, the concentrations of IFN-β, IFN-γ, IL-6, IL-12, and IL-27 were significantly higher in alveolar macrophages cultures from *L. rhamnosus* CRL1505-treated infant mice when compared to the control group. No differences were observed in IL-10 levels when control macrophages were compared to those obtained from CRL1505-treated infant mice (Figure 4). The levels of all the cytokines evaluated in alveolar macrophages cultures obtained from *L. plantarum* CRL1506-treated infant mice were not different from controls after poly(I:C) stimulation (Figure 3) or RSV challenge (Figure 4).

These results indicate that the signals delivered by orally administered *L. rhamnosus* CRL1505 to alveolar macrophages are capable of modulating the cytokine response of these immune cells in front of RSV infection and TLR3 activation.

### Effect of *L. rhamnosus* CRL1505 on Alveolar Macrophages Antiviral Factors Expression in Response to RSV Infection

**Figure 5:**
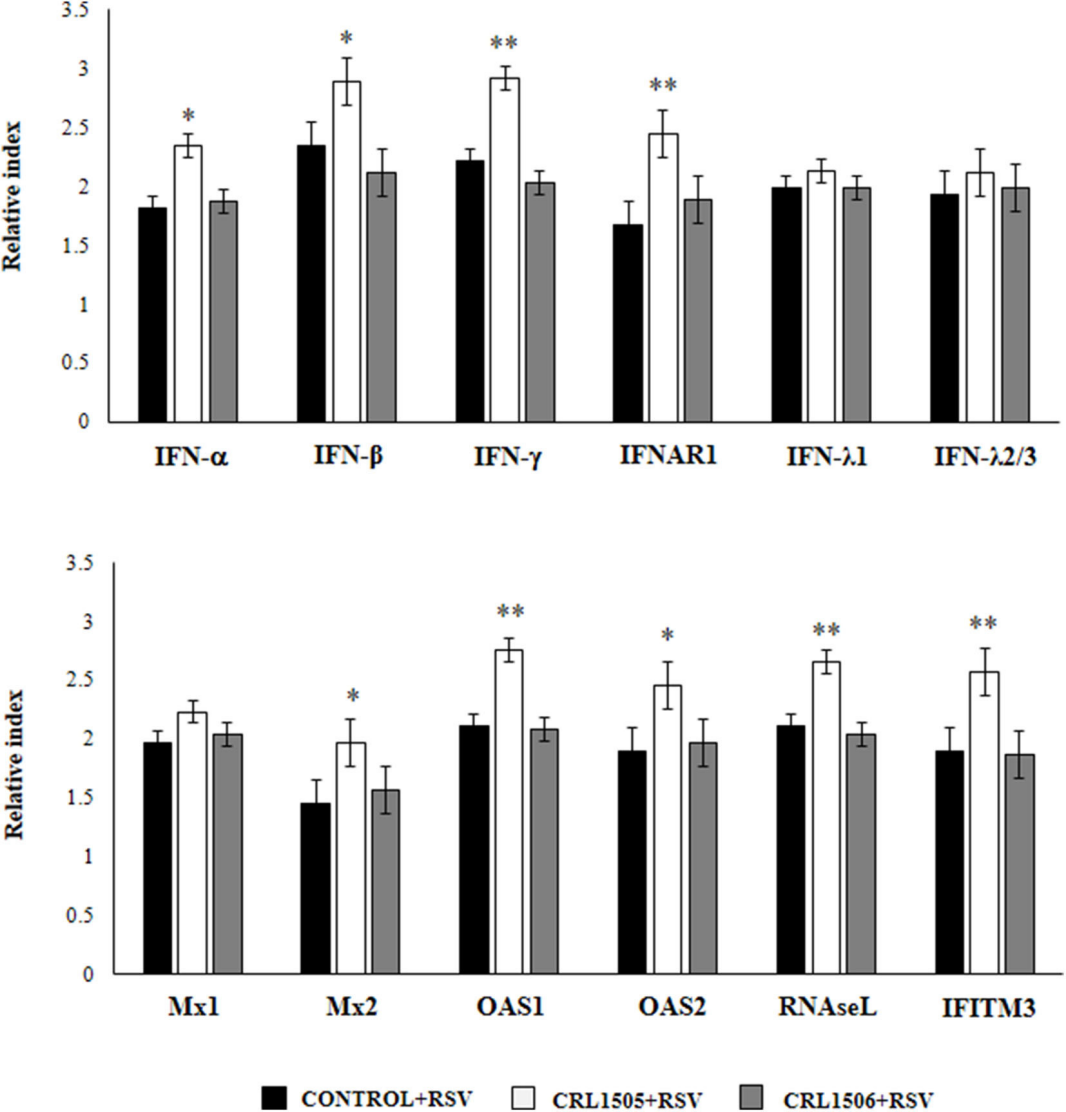
Effect of *Lacticaseibacillus rhamnosus* CRL1505 on alveolar macrophages antiviral factors profiles in response to Respiratory Syncytial Virus (RSV) infection. Infant mice were orally treated with *L. rhamnosus* CRL1505 or *Lactiplantibacillus plantarum* CRL1506 (10^8^ cell/mouse/day) during five consecutive days. One day after the last lactobacilli administration, alveolar macrophages were isolated from infant mice, cultured and challenged *in vitro* with RSV. Twelve hours after the viral infection, the expression of *IFN-*α*, IFN-*β*, IFN-*γ*, IFNAR1, IFN-*λ*1, IFN-*λ*2/3, Mx1, Mx2, OAS1, OAS2, RNAseL*, and *IFITM3* genes were evaluated by qRT-PCR. The results represent data from three independent experiments. Asterisks indicate significant differences when compared to the control group. *P < 0.05, **P < 0.01.

We further characterized the response of alveolar macrophages to RSV challenge by studying the expression levels of *IFN-*α*, IFN-*β*, IFN-*γ*, IFNAR1, IFN-*λ*1, IFN-*λ*2/3, Mx1, Mx2, OAS1, OAS2, RNAseL*, and *IFITM3* genes. Primary cultures from alveolar macrophages from control and lactobacilli-treated mice were stimulated with RSV (Figure 5). As expected, the expressions of *IFN-*α*, IFN-*β, and *IFN-*γ in alveolar macrophages from *L. rhamnosus* CRL1505-treated mice were significantly higher than the observed in macrophages from controls or mice treated with *L. plantarum* CRL1506. In accordance with the improved type I IFNs response, the levels of most of the ISGs in macrophages from the CRL1505 group were significantly higher than the control and the CRL1506 groups including *IFNAR1, Mx2, OAS1, OAS2, RNAseL*, and *IFITM3* (Figure 5). The only exception was *Mx1*, which expression levels were similar in all the experimental groups. In addition, no differences were found between control and lactobacilli-treated mice when the expression of *IFN-*λ*1* and *IFN-*λ*2/3* were compared. These results indicate that the signals delivered by orally administered *L. rhamnosus* CRL1505 to alveolar macrophages not only modulate the cytokine response of these immune cells in front of RSV infection but also in addition increase their antiviral state.

### Induction of Activated Alveolar Macrophages by *L. rhamnosus* CRL1505

**Figure 6:**
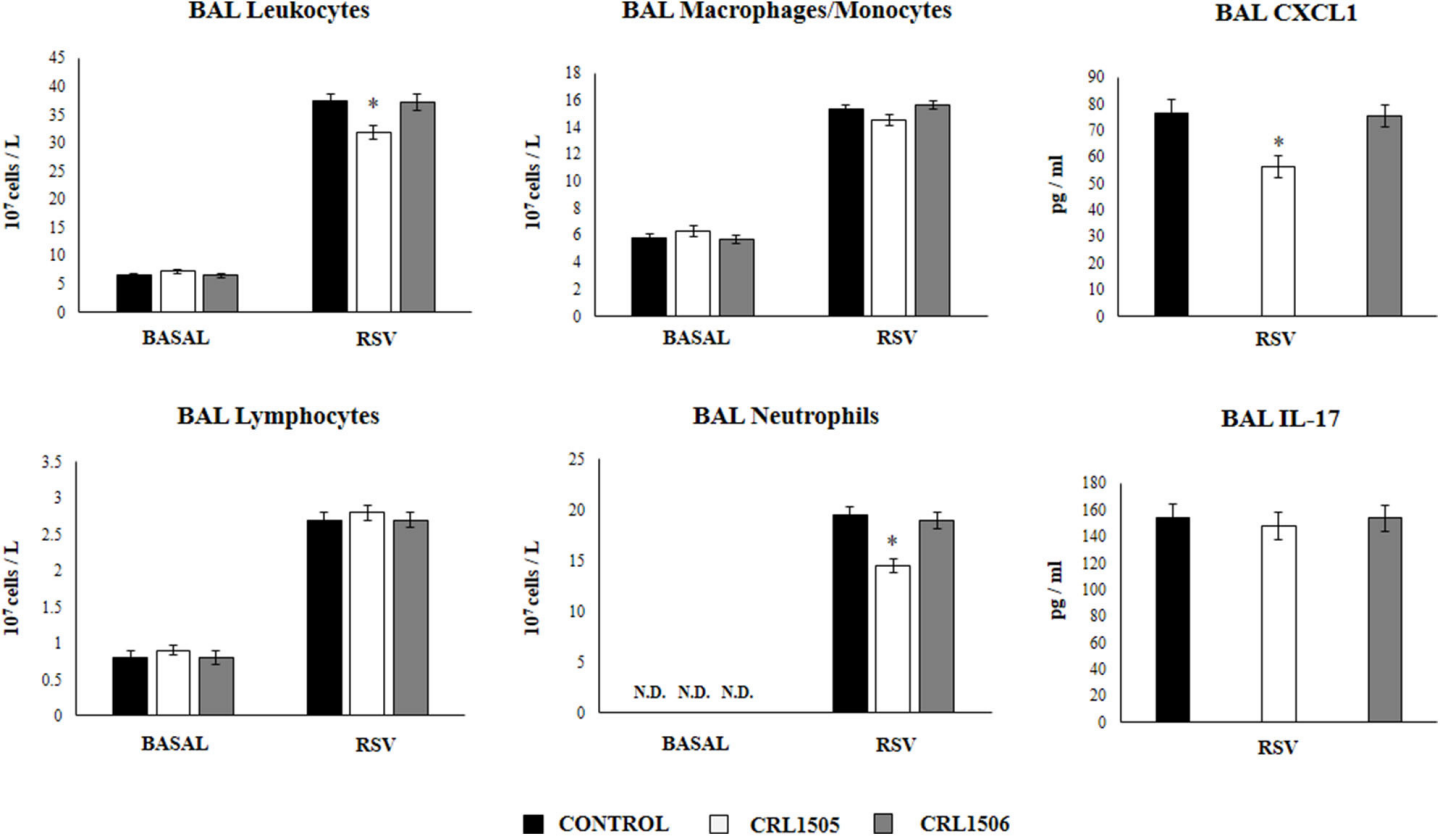
Effect of *Lacticaseibacillus rhamnosus* CRL1505 on broncho-alveolar lavages (BAL) leucocytes variations and CXCL1 and IL-17 levels in response to Respiratory Syncytial Virus (RSV) infection. Infant mice were orally treated with *L. rhamnosus* CRL1505 or *Lactiplantibacillus plantarum* CRL1506 (10^8^ cell/mouse/day) during five consecutive days. One day after the last lactobacilli administration, mice were challenged with RSV. The numbers of BAL leucocytes, neutrophils, lymphocytes, and macrophages/monocytes were evaluated before (basal) and 2 days after the RSV infection. The levels of BAL CXCL1 and IL-17 were measured 2 days after RSV challenge. The results represent data from three independent experiments. Asterisks indicate significant differences when compared to the control group. *P < 0.05.

We next aimed to evaluate the influence of the CRL1505 strain on the numbers and activation of immune cell populations in BAL before and after RSV infection. Alveolar macrophages are the main cell population in BAL samples of non-infected mice while lymphocytes are a minor population and neutrophils are not detected (Figure 6). *L. rhamnosus* CRL1505 or *L. plantarum* CRL1506 treatments did not modify the numbers of BAL leucocytes in the steady state. The challenge with RSV significantly increased the numbers of all BAL immune cell populations in the three experimental groups. However, CRL1505-treated mice had significantly lower numbers of total leukocytes as well as neutrophils counts when compared to controls (Figure 6). In line with this finding, the levels of CXCL1 in BAL samples from CRL1505-treated mice were significantly lower than controls after RSV infection. Of note, BAL IL-17 levels were not different when control and CRL1505-treated animals were compared (Figure 6). *L. plantarum* CRL1506 treatment was not able to modify the levels of BAL leukocytes or the concentrations of CXCL1 or IL-17 after the challenge with RSV with respect to the control group.

**Figure 7:**
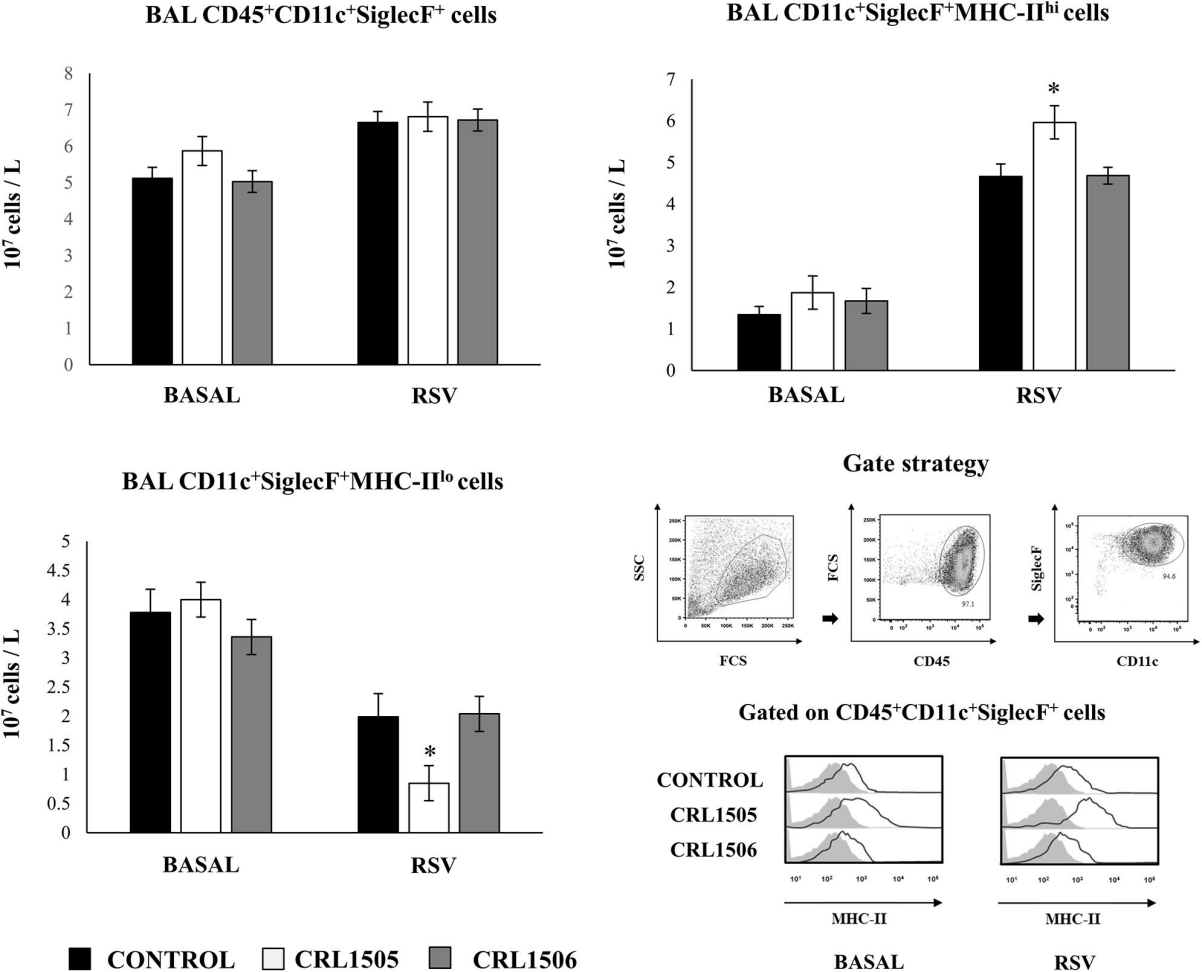
Effect of *Lacticaseibacillus rhamnosus* CRL1505 on resident alveolar macrophages number and activation in response to Respiratory Syncytial Virus (RSV) infection. Infant mice were orally treated with *L. rhamnosus* CRL1505 or *Lactiplantibacillus plantarum* CRL1506 (10^8^ cell/mouse/day) during five consecutive days. One day after the last lactobacilli administration, mice were challenged with RSV. Total resident alveolar macrophages populations in broncho-alveolar lavages (BAL) (CD45^+^CD11c^+^SiglecF^+^ cells) as well as their expression of MHC-II were evaluated before (basal) and 2 days after the RSV infection. The results represent data from three independent experiments. Asterisks indicate significant differences when compared to the control group. *P < 0.05.

We further characterized the variations of resident alveolar macrophages in BAL samples by flow cytometry. Total resident alveolar macrophages population in BAL (CD45^+^CD11c^+^SiglecF^+^ cells) were evaluated 1 day after lactobacilli treatments, and 2 days after the infection with RSV (Figure 7). The total numbers of alveolar macrophages were not modified by lactobacilli treatments in the steady state. The numbers of CD45^+^CD11c^+^SiglecF^+^ cell slightly increased upon the challenge with RSV (Figure 7) indicating that the increase of total numbers of BAL monocytes/macrophages (Figure 6) were mainly produced by recruited cells. No differences were found in CD45^+^CD11c^+^SiglecF^+^ cells between lactobacilli-treated and control mice (Figure 7). The CD11c^+^SiglecF^+^MHC-II^hi^ alveolar macrophages population was also evaluated in BAL samples. In control mice, CD11c^+^SiglecF^+^MHC-II^hi^ cells represented around the 25% of the total resident alveolar macrophages population, being most alveolar macrophages MHC-II^lo^ cells. The number of CD11c^+^SiglecF^+^MHC-II^hi^ cells significantly increased after the RSV infection (Figure 7). The treatment with *L. rhamnosus* CRL1505 did not induced statistical significant modifications in the numbers of CD11c^+^SiglecF^+^MHC-II^hi^ cells at basal time, although the MFI of MHC-II expression in alveolar macrophages of this group of mice was higher than controls (Figure 7). In addition, the numbers of CD11c^+^SiglecF^+^MHC-II^hi^ cells in *L. rhamnosus* CRL1505-treated mice were higher than controls after the RSV infection while CD11c^+^SiglecF^+^MHC-II^lo^ cells were reduced (Figure 7). MHC-II^hi^ and MHC-II^lo^ resident alveolar macrophages in mice treated with *L. plantarum* CRL1506 were not different from controls in the two time points evaluated (Figure 7). Then, these results allow us to speculate that the *L. rhamnosus* CRL1505 treatment would be able to differentially regulate the recruitment of immune cells into the respiratory tract in response to RSV infection by modulating the activation of alveolar macrophages.

### Role of CD4 Cells and IFN-γ in the Modulation of Alveolar Macrophages by *L. rhamnosus* CRL1505

**Figure 8:**
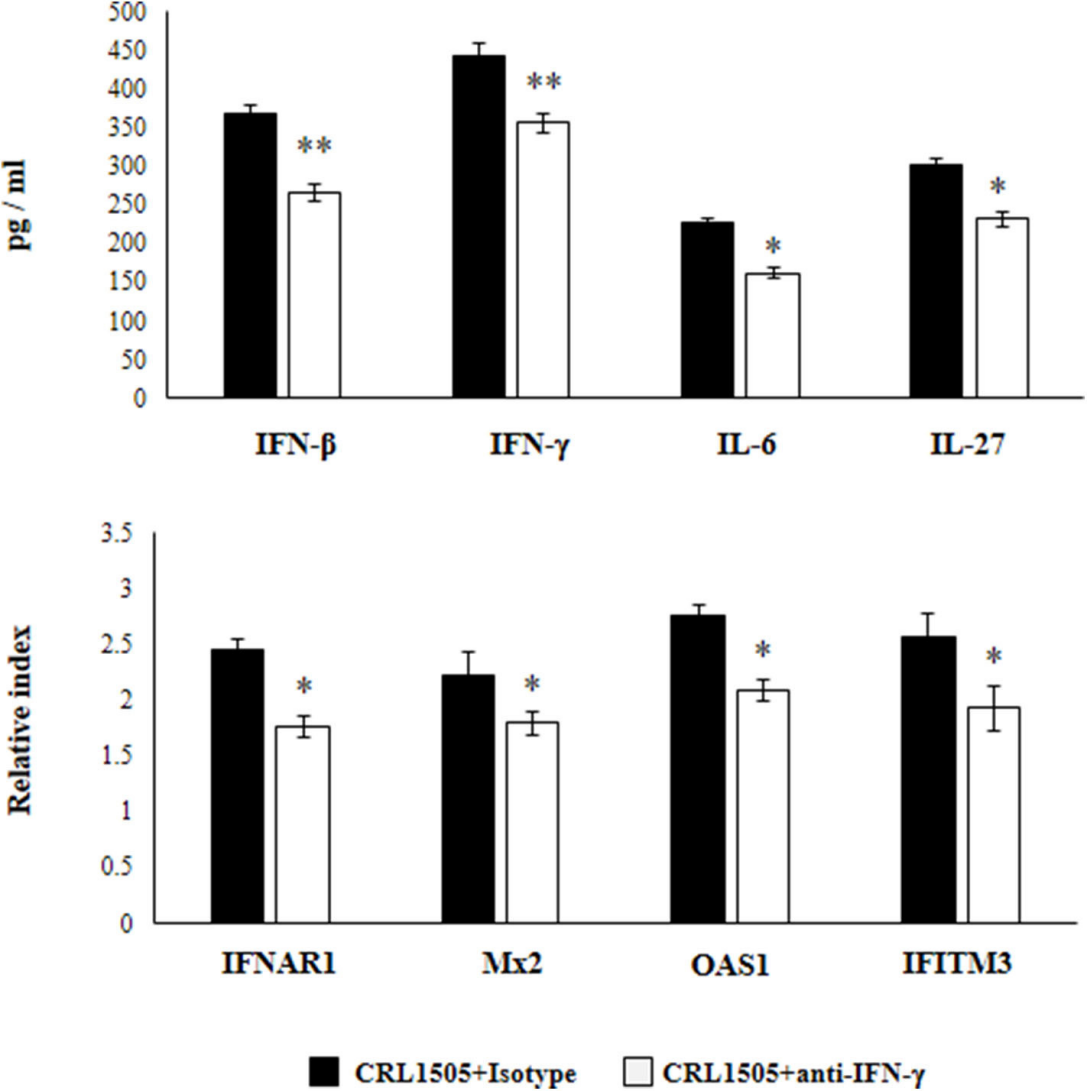
Effect of interferon (IFN)-γ depletion on the ability of *Lacticaseibacillus rhamnosus* CRL1505 to modulate the respiratory immune response triggered by Respiratory Syncytial Virus (RSV) infection. Infant mice were orally treated with *L. rhamnosus* CRL1505 (10^8^ cell/mouse/day) during five consecutive days. On days 1, 3, and 5 mice were injected intraperitoneally with purified anti-IFN-γ blocking antibodies or isotype control antibodies. One day after the last lactobacilli administration, alveolar macrophages were isolated from infant mice, cultured, and challenged *in vitro* with RSV. Twelve hours after the viral infection, the expression of *IFNAR1, Mx2, OAS1*, and *IFITM3* genes were evaluated by qRT-PCR. Twenty-four hours after the viral infection, the levels of interferon IFN-β, IFN-γ, interleukin (IL)-6, and IL-27 were evaluated on alveolar macrophages supernatants. The results represent data from three independent experiments. Asterisks indicate significant differences when compared to the isotype control group. *P < 0.05, **P < 0.01.

Previously, we demonstrated that the use of anti-IFN-γ blocking antibodies abolish the ability of the CRL1505 strain to reduce RSV titers in infected infant mice. In addition, the blocking of IFN-γ partially affected the reduction of the lung inflammatory damage during the course of RSV infection induced by orally administered *L. rhamnosus* CRL1505 (13). Then, we aimed to evaluate whether IFN-γ was related to the effect of the immunobiotic strain on alveolar macrophages. For this purpose, anti-IFN-γ blocking antibodies were administered to mice during the oral treatment with *L. rhamnosus* CRL1505. Primary cultures from alveolar macrophages obtained from these mice and isotype-treated control animals were prepared and stimulated *in vitro* with RSV (Figure 8). The production of IFN-β was significantly lower in alveolar macrophages isolated from anti-IFN-γ blocking antibodies-treated mice when compared to isotype controls. Consistently with this finding, it was observed a significant reduction in the expression of *IFNAR1, Mx2, OAS1*, and *IFITM3* genes in alveolar macrophages from anti-IFN-γ blocking antibodies-treated mice than in isotype controls (Figure 8). In addition, a significant reduction of IFN-γ, IL-6, and IL-27 production was observed in alveolar macrophages of mice depleted from IFN-γ during *L. rhamnosus* CRL1505 administration (Figure 8).

**Figure 9:**
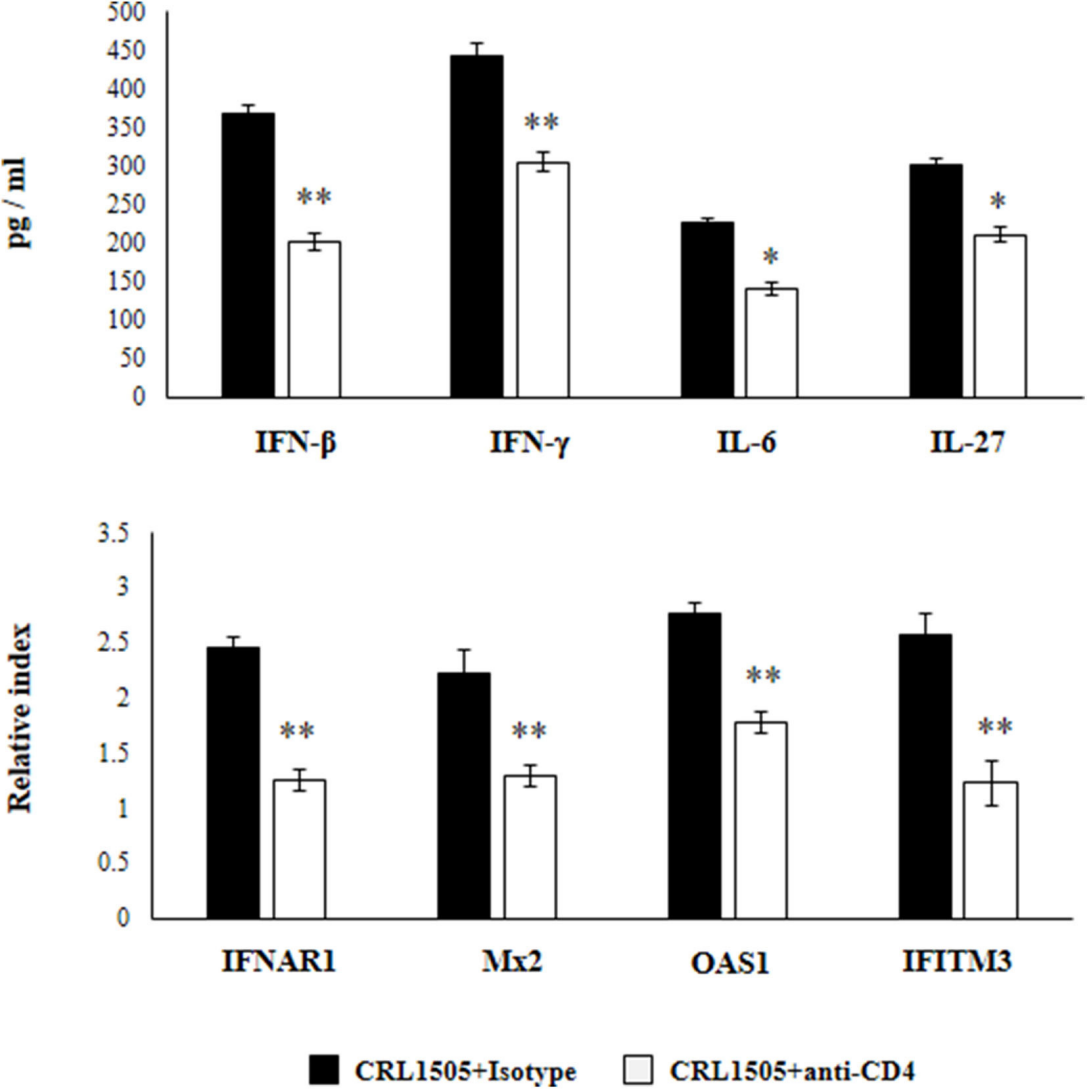
Effect of CD4 T cells depletion on the ability of *Lacticaseibacillus rhamnosus* CRL1505 to modulate the respiratory immune response triggered by Respiratory Syncytial Virus (RSV) infection. Infant mice were orally treated with *L. rhamnosus* CRL1505 (10^8^ cell/mouse/day) during five consecutive days. On days 1, 3, and 5 mice were injected intraperitoneally with purified anti-CD4 blocking antibodies or isotype control antibodies. One day after the last lactobacilli administration, alveolar macrophages were isolated from infant mice, cultured, and challenged *in vitro* with RSV. Twelve hours after the viral infection, the expression of *IFNAR1, Mx2, OAS1*, and *IFITM3* genes were evaluated by qRT-PCR. Twenty-four hours after the viral infection, the levels of interferon IFN-β, IFN-γ, interleukin (IL)-6, and IL-27 were evaluated on alveolar macrophages supernatants. The results represent data from three independent experiments. Asterisks indicate significant differences when compared to the isotype control group. *P < 0.05, **P < 0.01.

In another set of experiments, anti-CD4 blocking antibodies were administered to mice during the oral treatment with *L. rhamnosus* CRL1505. The response of primary cultures of alveolar macrophages of this group of mice to RSV challenge was also evaluated (Figure 9). Similar to IFN-γ blocking experiments, the depletion of CD4 cells during *L. rhamnosus* CRL1505 administration significantly reduced the capacity of alveolar macrophages to produce IFN-β, IFN-γ, IL-6, and IL-27 or express *IFNAR1, Mx2, OAS1*, and *IFITM3* genes in response to RSV challenge (Figure 9). Interestingly, anti-CD4 blocking antibodies induced a significantly higher reduction of IFN-β, IFN-γ, and ISGs than anti-IFN-γ blocking antibodies.

These results highlight the role of IFN-γ and CD4 cells in the modulation of alveolar macrophages activation induced by orally administered *L. rhamnosus* CRL1505.

## Discussion

Studies reported an important role for intestinal microbiota in maintaining respiratory antiviral immunity through the modulation of the immune response both at the steady state as well as in response to the viral attack (1–3). Remarkably, research work showed a significant impact of the intestinal microbiota in the respiratory innate antiviral defense mechanisms thought its influence on antigen presenting cells. The studies of Abt et al. (2) demonstrated that the intestinal microbiota help to maintain the optimal functions of pulmonary macrophages. The intestinal microbiota is involved in the efficient capacity of pulmonary macrophages to produce type I IFNs, IFN-λ and antiviral factors including *Irf7, Ifngr1, Stat1, Stat2, Mda-5, Rig-I, Ifit3, Mx1*, and *Oas1* to limit IFV replication (2). In line with these findings, some studies reported the ability of orally administered immunobiotic strains to regulate the function of macrophages in the respiratory tract. It was shown that the oral treatment of adult mice with *L. gasseri* A5 differentially regulated the cytokine profile of alveolar macrophages by increasing their production of IL-12 and decreasing IL-17 and IL-23 (23). The oral administration of a complex mixture of probiotic strains, which included *L. rhamnosus* GG and *E. coli* Nissle 1917 among others, was able to modify the composition of the intestinal microbiota and their metabolic profiles inducing an augmented production of short chain fat acids. These metabolic products influenced alveolar macrophages' function by up-regulating their expression of IFN-β and antiviral factors, increasing the resistance of adult mice to RSV infection (24). In addition, feeding of adult mice with *L. gasseri* SBT2055 improved the expression of *Mx1* and *Oas1* in alveolar macrophages and diminished the susceptibility to IFV infection (25). An enhanced resistance to IFV challenge was also observed in adult mice orally treated with *L. plantarum* DK119. Interestingly, the depletion of alveolar macrophages completely abolished the capacity of the DK119 strain to protect mice against the respiratory viral infection (26).

In line with those studies, we previously reported that orally administered *L. rhamnosus* CRL1505 was capable to modulate alveolar macrophages function in adult mice (27). The CRL1505 strain increased the phagocytic and microbicidal activity of alveolar macrophages, and improved their production of IFN-γ and TNF-α in response to *Streptococcus pneumoniae* challenge. In addition, increased numbers of CD11c^+^SiglecF^+^IFN-β^+^ alveolar macrophages were found in mice preventively treated with *L. rhamnosus* CRL1505 and then challenged with poly(I:C) or RSV (13, 14). In this work, we have extended those previous findings by consistently demonstrating a relevant role of alveolar macrophages in the immunomodulatory capacity of orally administered *L. rhamnosus* CRL1505 in the context of RSV infection in infant mice. Macrophages depletion experiments and a detailed study of their production of cytokines and antiviral factors clearly demonstrated the key role of this immune cell population in the improvement of both viral elimination and protection against lung tissue damage induced by the immunobiotic CRL1505 strain.

The phagocytic activity of alveolar macrophages is crucial for the elimination of infected cells during the course of respiratory virus infection. In addition, it was reported that alveolar macrophages play a prominent role in the defense against respiratory viruses such as RSV by producing type I IFNs (28). Moreover, some studies suggested that alveolar macrophages are the most important producers of IFN-α/β in response to RSV challenge, even when compared to other respiratory cells such as epithelial cells and plasmacytoid DCs (29, 30). Type I IFNs produced by alveolar macrophages can act on this same cell population or on other immune and non-immune cells of the respiratory tract modulating their expression of hundreds of ISGs that contribute to viral clearance (31). Type I IFNs production by alveolar macrophages also induce the expression of several monocyte chemoattractants in the respiratory tract that induce the recruitment of CD11c^lo^CD64^hi^CD11b^+^ inflammatory monocytes/macrophages that further support the clearance of virus-infected cells (29). On the other hand, it was reported that RSV is capable of infecting both human and murine alveolar macrophages although the infection is abortive since there is no increment of viral particle production (18, 32). However, RSV induce a significant impairment in the production of IFN-γ and IL-12 by alveolar macrophages (33) that was associated with an enhanced severe illness in infants (34). The reduced levels of IFN-γ diminish alveolar macrophages activation, impairing their phagocytic function and their ability to induce the recruitment of T and NK cells to the lungs, contributing to higher viral replication (35, 36). Then, the efficient and timely production of type I IFNs, ISGs and IFN-γ is important to confer protection against RSV.

We previously reported enhanced levels of IFN-β and IFN-γ in BAL samples of CRL1505-treated mice after TLR3 activation (13), RSV challenge (14) or IFV infection (37). The results of this work clearly demonstrated the ability of orally administered *L. rhamnosus* CRL1505 to enhance the ability of alveolar macrophages to produce type I IFNs, ISGs and IFN-γ in response to RSV infection. Our results indicate that alveolar macrophages greatly contribute to the augment of IFN-β and IFN-γ in the respiratory tract of CRL1505-treated mice. Moreover, we reported here for the first time an improved expression of *IFNAR1, Mx2, OAS1, OAS2, RNAseL*, and *IFITM3* in alveolar macrophages after the oral treatment with *L. rhamnosus* CRL1505. OAS1 is capable of inhibiting protein synthesis and viral growth by degrading viral and cellular RNA while IFITM3 has the ability to block early events in the viral replication cycle. Both, OAS1 and IFTIM3 have been shown to interfere with RSV replication and limit productive infection (31, 38). The members of OAS family are able to activate RNAseL, being AOS2 more efficient that OAS1 to induce this effect. The intracellular endoribonuclease RNaseL activated by OAS molecules cleaves viral and cellular RNA resulting in apoptosis (39). It was shown that IFN-γ is able to up-regulate the activities of OAS/RNAseL increasing the protection against RSV infection (40). In addition, recent studies in cotton rats demonstrated differences in RSV infection severity related to the age. While adult animals were resistant to RSV infection, infant rats were highly susceptible to the viral infection. Interestingly, the work reported that the higher viral load and lung pathology observed in younger animals was related to a lower ability to up-regulate IFN-α/Mx2 levels in the respiratory tract (41). The enhancement of these antiviral factors is consistent with the improved clearance of RSV (14) and IFV (37) induced by the oral treatment with the immunobiotic strain. Furthermore, the identification of the differential antiviral factors and cytokines profiles induced by *L. rhamnosus* CRL1505 in alveolar macrophages indicate that the immunobiotic treatment has the potential to protect against other respiratory viruses as well.

The role of alveolar macrophages in the protective immune responses against RSV have been demonstrated in some animal models in which this immune cell population was specifically depleted. Experiments in adult mice demonstrated that the depletion of alveolar macrophages by the administration of clodronate liposomes before the challenge with RSV significantly impaired the production of IFN-α, TNF-α, and IL-6 in the respiratory tract and diminished the activation and recruitment of NK cells. Those changes were associated to an enhanced lung RSV load (42). Experiments in CD169-diphtheria toxin receptor transgenic mice, which are depleted from CD169^+^ alveolar macrophages after the administration diphtheria toxin, also demonstrated that macrophages elimination impaired the production of IFN-β, IL-6, and TNF-α in the respiratory tract in response to RSV infection (43). By using a similar approach, we demonstrated here that the depletion of alveolar macrophages by the time of *L. rhamnosus* CRL1505 administration abolished the ability of infant mice to produce improved levels of IFN-β in response to TLR3 activation or RSV infection. In addition, although the production of IFN-γ was diminished when alveolar macrophages were depleted; the levels of this cytokine were still significantly higher in CRL1505-treated mice than in controls. These results indicate that other immune cell population also contribute to the improved levels of IFN-γ in the respiratory tract of CRL1505-treated mice. The most likely source of IFN-γ are CD4+ T cells as discussed below. Of note, the depletion of alveolar macrophages completely abolished the ability of orally administered *L. rhamnosus* CRL1505 to improve IL-10 in the respiratory tract or to reduce the biochemical markers of lung injury after TLR3 activation or RSV infection. Then, alveolar macrophages had a key role in the protection against the lung detrimental inflammation induced by the immunobiotic CRL1505 strain.

The alteration of alveolar macrophages function by the infection with RSV has been associated to an exacerbated viral-mediated bronchiolitis (35). In fact, the depletion of alveolar macrophages greatly increased the recruitment of inflammatory cells to the lung during the early stage of RSV infection. The depletion of alveolar macrophages induce a significant greater lung inflammation in response to RSV challenge that is characterized by increases in CD11b^hi^Gr1^hi^ neutrophils and inflammatory CD11c^hi^MHC-II^hi^CD11b^+^ DCs that contribute to an hyperresponsiveness in RSV-infected mice (18). Notably, this deregulated inflammatory response contribute poorly to the elimination of the virus while promoting local damage and affecting lung function. Then, the immunoregulatory functions of alveolar macrophages seems crucial for avoiding the lung inflammatory-mediated damage during the course of RSV infection. Several mechanisms have been proposed for the immunoregulatory functions of alveolar macrophages in the context of viral infections. The most obvious function of macrophages is the phagocytosis of virus-infected apoptotic cells preventing the release of cellular contents to local environment and avoiding the triggering of further inflammatory factors production (44). It was also shown that alveolar macrophages produce anti-inflammatory cytokines such as IL-10, especially during the resolution of the infection (32, 44). Another anti-inflammatory strategy of alveolar macrophages is their ability to promote Treg cells responses by directly interacting with these cells or indirectly through the production of certain cytokines (45, 46). It was reported that resident CD11c^+^SiglecF^+^ alveolar macrophages pulsed *in vitro* with ovalbumin are able to induce the development of Foxp3^+^ Treg cells when they are co-cultured with antigen-specific CD4 T cells (45). Moreover, the transfer of the ovalbumin-pulsed macrophages into the respiratory tract of mice significantly reduced the lung inflammation upon subsequent stimulation with the antigen. On the other hand, it was demonstrated that IL-6 is required for the reduction of RSV-induced immunopathology. The early production of IL-6 after RSV challenge stimulates the expression of IL-27 by alveolar macrophages, which in turn promotes the maturation of Treg cells in the respiratory tract (46). Our previous studies (13, 14, 37) and the results presented here allow us to conclude that some of these anti-inflammatory functions of alveolar macrophages are enhanced by the oral administration of *L. rhamnosus* CRL1505.

We have reported increased levels of IL-10 in the respiratory tract of *L. rhamnosus* CRL1505-treated mice and partially attributed this increment to the enhanced production of this immunoregulatory cytokine by alveolar macrophages (13, 14, 27). The data of this work show that this assertion was incorrect, since the study of the production of IL-10 by the alveolar macrophages in CRL1505-treated infant mice were not different from controls at the basal level or upon stimulation with poly(I:C) or RSV. On the other hand, we demonstrated here for the first time that alveolar macrophages from *L. rhamnosus* CRL1505-treated mice had a significantly increased capacity to produce IL-27 in response to TRL3 activation or RSV stimulation. The immunoregulatory cytokine IL-27 have been shown to modulate inflammatory responses by acting in cells from both innate and adaptive immunity (47). Recent studies highlighted the role of IL-27 in the protection against lung inflammatory damage during the course of viral infections. It was reported that IL-27RA^−/−^ mice have an exaggerated lung immunopathology after the infection with IFV, that correlated with increased levels of CD4^+^ and CD8^+^ T cells producing IL-17 and a strong neutrophil infiltration (48). In addition, the depletion of IL-27 in the respiratory tract during RSV infection enhanced the damaging inflammation (46). It was also shown that IL-27 helps in the control of RSV infection severity by suppressing Th17- and Th2-mediated inflammation (49). Interestingly, comparative studies of IFV infection in IL-27RA^−/−^ and IL-10^−/−^ mice demonstrated that the former had a more severe disease course than the latter (48), demonstrating that not all the anti-inflammatory effects of IL-27 are mediated by the induction of IL-10 production as it has been suggested (47, 50, 51). Of note, it was reported that IL-27 is not sufficient for the optimal induction of Treg cells maturation in the respiratory tract and that IL-6 is required for the IL-27/Treg cells protection against inflammatory damage. The early production of IL-6 after RSV infection induce the production of IL-27 by myeloid cells including alveolar macrophages, which in turn stimulates Treg cell maturation. Depletion of IL-27 or IL-6 in the respiratory tract during RSV have the same detrimental effect on the maturation of Treg cells and RSV-mediated immunopathology (46). Then, our results show that the improved production of IL-27 and IL-6 by alveolar macrophages of CRL1505-treated mice may play an important role in limiting inflammation and protecting lung function during RSV infection, by increasing the maturation and activation of Treg cells.

Macrophage's functions have been studied in the context the M1/M2 polarization dichotomy being classically activated macrophages (M1-like) associated to pro-inflammatory microenvironments while alternatively activated macrophages (M2-like) developed in anti-inflammatory environments (52). M1-like macrophages are characterized by a high phagocytic activity and by their ability to guide acute inflammatory responses through their production of pro-inflammatory factors that stimulate the Th1 response. On the other hand, M2-like macrophages negatively regulate pro-inflammatory cytokines, and induce the production of anti-inflammatory mediators contributing to the control of inflammation, tissue repair and the return to hemostasis of the infected tissue (52). However, recent advances in the biology of macrophages have demonstrated that this dichotomy separating M1-like and M2-like macrophages is rather represented by a continuum between the two states. This fact is particularly true for alveolar macrophages that have been shown to express a combined M1/M2 phenotype (53). It was suggested that this hybrid phenotype confer to alveolar macrophages the ability to quickly switch between M1 or M2 associated functions allowing for appropriate responses to stimuli and tissue environment. During the acute phase of inflammation, alveolar macrophages may function as M1 activated cells inducing the activation of various mechanisms that contribute to pathogen elimination. However, such responses must be controlled to prevent lung tissue damage through the activation of M2 functions that control inflammatory reactions, and promote and accelerate the wound healing process and tissue repair. Such a fine-tuned balance and switching back and forth between the M1 and M2 polarization states in alveolar macrophages would be necessary to allow the beneficial processes of inflammation, resolution, and repair (52, 53). Then, it could be concluded that the oral treatment with *L. rhamnosus* CRL1505 would not induce differential modifications of alveolar macrophages but instead it would stimulate and improve a function that is already programmed in this particular population of immune cells of the respiratory tract.

As mentioned before, studies demonstrated that alveolar macrophages have a great impact in the innate antiviral immune response to RSV. However, their role in the generation of adaptive immune responses has not been completely elucidated. It was shown the macrophage depletion did not affect the recruitment of activated CD4^+^ T cells into the lung, indicating that this immune cell population may have little effect on the adaptive response to RSV (42). In contrast, the depletion of alveolar macrophages before the nasal immunization with an experimental RSV vaccine based on the viral G protein significantly reduced the levels of specific neutralizing antibodies (54). Mice with depleted alveolar macrophages had an impaired protection against RSV challenge when compared to normal controls. In addition, the depletion of CD169^+^ alveolar macrophages reduced the recruitment of effector CD8^+^ T cells to the lungs after RSV infection (43). On the other hand, it was reported that the influence of intestinal microbiota on the respiratory innate antiviral immune response is able to condition the subsequent adaptive immune responses. The intestinal microbiota was shown to positively influence the ability of pulmonary macrophages to support the generation of virus-specific antibodies as well as virus-specific T cells. In intestinal microbiota-depleted mice, macrophages had a reduced expression of MHC-I and CD86 molecules. An impaired number of IFV-specific CD8^+^ T cells as well as a reduced ability of these cells to produce IFN-γ, TNF-α, IL-2, and MIP-1α was also observed (2). Taking into consideration the findings of this work demonstrating that orally administered *L. rhamnosus* CRL1505 influence the MHC-II expression of the alveolar macrophages during the course of RSV infection, it is tempting to speculate that the immunobiotic intervention would also beneficially modify the adaptive immune response against the viral pathogen in infant mice. In support of this hypothesis, it has been shown that IFN-γ is able to up-regulate MHC-II expression in alveolar macrophages improving their antigen presentation activities (55). The evaluation of the influence of *L. rhamnosus* CRL1505 in the adaptive immune response to RSV and the role of alveolar macrophages in this potential beneficial effect is an interesting topic for future research.

In addition, we provided evidence that support an important role of CD4^+^ cells and IFN-γ in the ability of *L. rhamnosus* CRL1505 to modulate alveolar macrophages activities and the subsequent improved response to RSV infection. In our hands, the administration of anti-IFN-γ or anti-CD4 antibodies at the time of the CRL1505 treatment completely abolished the ability of the immunobiotic strain to differentially modulate the cytokine and antiviral factors profile of alveolar macrophages. The inefficient production of IFN-γ in the respiratory tract during the course of RSV infection with the subsequent impaired activation of alveolar macrophages has been associated with an increase of severe bronchiolitis and pneumonia in neonates and infants (34–36). In addition, comparative studies of RSV challenge in infant and adult mice demonstrated that in IFN-γ production were associated to the distinct susceptibility of both groups to viral infection (36, 55). Adult mice are able to induce the recruitment of CD8^+^IFN-γ^+^ and CD4^+^IFN-γ^+^ T cells in the lung, which activated alveolar macrophages and promoted an efficient viral clearance. In contrast, infant mice were capable of inducing the recruitment of CD8^+^IFN-γ^+^ T cells in the respiratory tract. The lack of effective recruitment of CD4^+^IFN-γ^+^ T cells into the alveolar space and the lower activation of macrophages correlated with the higher RSV loads in the lungs of infant mice. These previous findings and the results of this work highlight the role of CD4^+^ cells and IFN-γ in the activation of alveolar macrophages induced by *L. rhamnosus* CRL1505 and in the modulation of respiratory antiviral immunity.

The exact origin and nature of the signals used by the intestinal microbiota or orally administered immunobiotics to modulate the respiratory antiviral immunity remain to be determined. Our experiments blocking CD4^+^ cells and IFN-γ allow us to hypothesize about a potential mechanism that could explain the remote effect induced by orally administered *L. rhamnosus* CRL1505. The existence of the so-called common mucosal immune system implies that the immune cells activated in one mucosal tissue can mobilize and reach distant mucosal sites where they can influence immune responses. Then, the mobilization of B and T cells from the intestinal mucosa to the respiratory tract could be involved in the beneficial effects exerted by the intestinal microbiota or immunobiotics (6, 56). It was also hypothesized that immune factors such as cytokines and growth factors produced in the intestinal mucosa in response to microbiota stimulation, can be released to blood and act systemically or in other mucosal tissues (56, 57). We previously demonstrated that both orally administered *L. rhamnosus* CRL1505 and *L. plantarum* CRL1506 were capable of improving the levels of IFN-γ in the intestine and blood, while only the CRL1505 strain increased this immune factor in the respiratory tract, indicating that the IFN-γ was produced locally (13). In addition, our previous results indicated that the oral administration of *L. rhamnosus* CRL1505 induces an increase of CD4^+^IFN-γ^+^ T cells in the lungs, and effect that was not observed in CRL1506-treated mice (14). Then, considering those previous results and the ones obtained in this work it is tempting to speculate that *L. rhamnosus* CRL1505 would induce the mobilization CD4^+^IFN-γ^+^ T cells from the intestine to the lungs, and that the IFN-γ produced by mobilized CD4^+^ T cells would modulate the respiratory tract innate immune microenvironment leading to the activation of local immune cells such as alveolar macrophages. Conducting more in-depth studies to verify this hypothesis is a task that we intend to carry out in the immediate future.

Of note, other probable mechanisms have been proposed, which are not mutually exclusive, to explain the effect of the intestinal beneficial microbes on the respiratory antiviral immunity. There is evidence that some microbial-associated molecular patterns (MAMPs) derived from the intestinal microbiota can be adsorbed and transported to extraintestinal sites where they stimulate pattern recognition receptors (PRRs) expressed in non-immune and immune cells influencing the immune responses (58, 59). In addition, microbial metabolites that are adsorbed in the intestine have been associated to the differential modulation of respiratory immune responses. This effect has been called “metabolic reprograming” (60) and implies that metabolites such as circulating short-chain fatty acids (61), desaminotyrosine (5), docosahexanoic acid (62), or acetate (4) can reach innate immune cells in the respiratory tract and improve their responses to viral infections. Studying whether these mechanisms contribute to the immunomodulatory effect of *L. rhamnosus* CRL1505 is also an interesting topic for future research.

Alveolar macrophages are a crucial component of innate host defense in the respiratory tract and this immune cell population have been demonstrated to play a critical role limiting the severity of RSV-induced disease. In this study, we defined the importance of alveolar macrophages as a key players in the immunomodulatory and protective activities of orally administered *L. rhamnosus* CRL1505 in the context of RSV infection in infants. In addition, we provided evidence of the important role of CD4^+^ cells and IFN-γ in the activation of alveolar macrophages highlighting a putative pathway through which the intestinal and respiratory mucosa are communicated under the influence of *L. rhamnosus* CRL1505.

## Data Availability Statement

The raw data supporting the conclusions of this article will be made available by the authors, without undue reservation.

## Ethics Statement

This study was carried out in strict accordance with the recommendations in the Guide for the Care and Use of Laboratory Animals of the Guidelines for Animal Experimentation of CERELA. The CERELA Institutional Animal Care and Use Committee prospectively approved this research.

## Author Contributions

JV and HK designed the study and wrote the manuscript. VG-C, MT, FR, and MI did the laboratory work. VG-C and MI performed statistical analysis. HT, HK, and JV contributed to data analysis and interpretation. All authors read and approved the manuscript.

## Conflict of Interest

The authors declare that the research was conducted in the absence of any commercial or financial relationships that could be construed as a potential conflict of interest.

## Abbreviations

BAL: Broncho-alveolar lavages
CXCL1: Chemokine (C-X-C motif) ligand 1
CLP: Clodronate-containing liposomes
DMEM: Dulbecco's modified Eagle's medium
ELISA: Enzyme-linked immunosorbent assay
ELP: Empty liposomes
HRP: Horseradish peroxidase
IFV: Influenza Virus
IL: Interleukin
IFN: Interferon
IFNAR1: IFN-alpha/beta receptor alpha chain
Mx: IFN-induced GTP-binding protein Mx
ISGs: Interferon-stimulated genes
IFITM3: IFN-induced transmembrane protein 3
LDH: Lactate dehydrogenase
MRS: Man-Rogosa-Sharpe
MAMPs: Microbial-associated molecular patterns
MOI: Multiplicity of infection
OAS: 2'-5'-oligoadenylate synthetase
PRRs: Pattern recognition receptors
PBS: Phosphate buffer saline
RSV: Respiratory syncytial virus
RNAseL: Ribonuclease L
TLR3: Toll-like receptor 3.
