# Supplementary material for: Alveolar Macrophages Are Key Players in the Modulation of the Respiratory Antiviral Immunity Induced by Orally Administered Lacticaseibacillus rhamnosus CRL1505
Source: Front Immunol. 2020 Sep 29;11:568636. doi: 10.3389/fimmu.2020.568636 (PMC7550464; doi:10.3389/fimmu.2020.568636)
Supplement: Supplementary file 1 [file Table_1.DOCX]

**Supplementary Table 1.** Primer sequences used in this study.

| **Gene** | **Sense primer (5’-3’)** | **Antisense primer (5’-3’)** |
| --- | --- | --- |
| **IFN-α** | GACTCATCTGCTGCTTGGAATGCAACCCTCC | GACTCACTCCTTCTCCTCACTCAGTCTTGCC |
| **IFN-β** | TCTGGAGCATCTCTTGGATGGCAA | TCCAGCTCCAAGAAAGGACGAACA |
| **IFN-γ** | GAAAGCCTAGAAAGTCTGAATAACT | ATCAGCAGCGACTCCTTTTCCGCTT |
| **IFN-λ1** | GACGAGTACAGGCAGCTTCC | AGCATTGACCCTTAGGATCTTCTC |
|  |  |  |
| **IFN-λ2/3** | AGTGGAAGCAAAGGATTG | GAGATGAGGTGGGAACTG |
| **IFNAR1** | AGCGTCTGGAAATACCTGTGTC | CTCAGCCGTCAGAAGTACAAGG |
| **Mx1** | CAGAGGTCAGCAGGACATCC | TCGCTTGCACTCTGATGACT |
| **Mx2** | AGAGGTACCAAGTCTAAGGGCTCTGAG | AGACTCGAGCAAATGCCCTGCTGTACT |
| **OAS1** | AAAAGGAGGAGCCATGGCAGT | CTGAGCCCAAGGTCCATCAG |
| **OAS2** | CACATATGGGAAACTGGCTGACTGGAAACT | CCTCGAGATTGCACAGGATCTCTGAGAGG |
| **RNAseL** | AAGCTTCTCAGGATCGAATGTACCAAC | GAATTCTCTGTCAAAGTGCACTGGGAC |
| **IFITM3** | CTGAACATCAGCACCTTGGT | TTTTGGTGGTTATCAAGTGCACT |
